# Polymeric Micelles with pH-Responsive Cross-Linked Core Enhance In Vivo mRNA Delivery

**DOI:** 10.3390/pharmaceutics14061205

**Published:** 2022-06-06

**Authors:** Wenqian Yang, Pengwen Chen, Eger Boonstra, Taehun Hong, Horacio Cabral

**Affiliations:** Department of Bioengineering, Graduate School of Engineering, The University of Tokyo, 7-3-1 Hongo, Bunkyo-ku, Tokyo 113-8656, Japan; yang-wenqian462@g.ecc.u-tokyo.ac.jp (W.Y.); pengwenchen@g.ecc.u-tokyo.ac.jp (P.C.); eger@g.ecc.u-tokyo.ac.jp (E.B.); hongtokyo9296@gmail.com (T.H.)

**Keywords:** mRNA, polymeric micelles, cross-linking, cancer, nanomedicine

## Abstract

Messenger RNA (mRNA) is emerging as a promising therapeutic modality for a variety of diseases. Because of the fragility and limited intracellular access of mRNA, the development of delivery technologies is essential for promoting the applicability of mRNA-based treatments. Among effective nanocarriers, polymeric micelles loading mRNA by polyion complex (PIC) formation with block catiomers have the potential to meet the delivery needs. Since PICs are relatively unstable in in vivo settings, herein, we constructed mRNA-loaded micelles having pH-responsive cross-linked cores by complexing mRNA with *cis*-aconitic anhydride-modified poly(ethylene glycol)-poly(l-lysine) (PEG-pLL(CAA)) block copolymers. The micelles were stable at physiological pH (pH 7.4) but achieved the complete release of the mRNA at endosomal pH (pH 5.5–4.5). The cross-linking also enhanced the stability of the micelles against disassembly from polyanions and protected the loaded mRNA from degradation by nucleases. Thus, the cross-linked micelles increased the delivery of mRNA to cancer cells, promoting protein expression both in vitro and in vivo. Our results highlight the potential of PEG-pLL(CAA)-based micelles for mRNA delivery.

## 1. Introduction

Messenger RNA (mRNA)-mediated transfection, which offers in situ production of therapeutic proteins, is rising as an attractive modality for a variety of therapeutic applications, such as vaccination, genome editing and protein replacement [1,2,3,4]. Compared to plasmid DNA, mRNA does not require transportation through the nuclear membrane and prevents the integration into the genome of the host cell [5], which are substantial advantages for enhanced efficiency and safety. Nevertheless, intracellular access and rapid degradation of mRNA by nucleases remain major issues for maximizing the therapeutic benefits.

Delivery systems are central to the success of mRNA therapy [1,5]. Among effective mRNA delivery systems, non-viral vectors offer safety, low immunogenicity, and high loading capacity [6,7]. Especially, polyion complex (PIC) micelles, which are core-shell self-assemblies from block catiomers and oppositely charged ionomers, have shown high potential to protect mRNA from degradation and recognition by immune receptors [8,9,10]. However, the stability of PICs is undermined by polyion exchange from negatively charged proteoglycans on cell surfaces and in plasma [11]. Therefore, to improve the performance of mRNA-loaded micelles in vivo settings, major efforts have been dedicated to enhancing the PIC stability, such as introducing hydrophobic moieties in the core forming materials [12,13], engineering the flexibility of the polycation block [14] and using cationic moieties with multivalent interactions [15]. On the other hand, stimuli-responsive stabilization approaches of the PICs could offer effective protection of the mRNA outside the cells while releasing active mRNA upon sensing the environment in subcellular spaces [16,17,18,19,20]. Particularly, the difference in the pH of the extracellular environment (pH 7.4) and the acidic pH in endosomes (pH 6.5–4.5) [21] provides a useful trigger to generate smart stabilization strategies for mRNA-loaded PICs.

**Figure 1:**
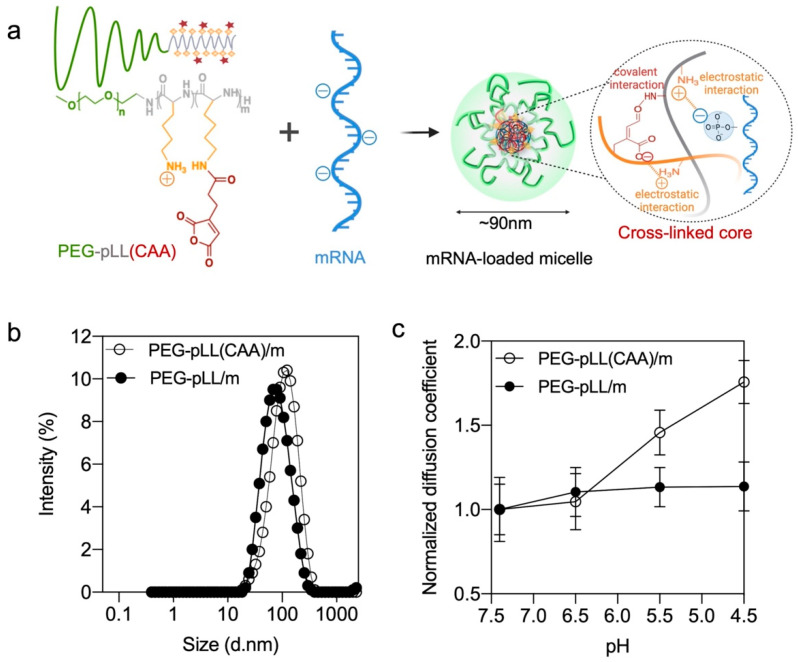
Micelle formation and characterization. (**a**) mRNA-loaded micelles formation based on electrostatic interaction and covalent interaction. (**b**) Size distribution of mRNA-loaded micelles by intensity (%) as determined by DLS. (**c**) Diffusion coefficient of micelles versus buffer pH (10 mM HEPES with 150 mM NaCl) having different pHs determined by FCS. The results are expressed as the mean ± s.d. (*n* = 10).

Herein, we developed mRNA-loaded PIC micelles stabilized by pH-responsive cross-linking of the core by employing *cis*-aconitic anhydride-modified poly(ethylene glycol)-poly(l-lysine) (PEG-pLL(CAA)) block copolymers. The residual amino groups in the pLL(CAA) segment can promote ion complexation with mRNA, while the reaction between the CAA moieties with the non-complexed primary amines can cross-link the core by forming pH-sensitive amide bonds (Figure 1a) [22]. The pH sensitivity and the stability of the cross-linked mRNA-loaded micelles were tested in vitro. Micelles prepared from PEG-pLL were used as control. Moreover, the ability of the micelles to deliver mRNA and produce proteins was evaluated in cancer cells, as well as in solid tumors in vivo. The results demonstrated that PEG-pLL(CAA)-based micelles can improve mRNA delivery into the cells to promote high levels of protein expression both in vitro and in vivo.

## 2. Materials and Methods

### 2.1. Materials

α-Methoxy-ω-amino poly(ethylene glycol) (MeO-PEG-NH_2_) (*M_w_* = 12,000 g mol^−1^) was purchased from NOF CORPORATION (Tokyo, Japan). ξ-Trifluoroacetyl-l-lysine *N*-carboxyanhydride (Lys-(TFA)-NCA) was purchased from Chuo Kaseihin Co. Inc. (Tokyo, Japan). *N*,*N*-dimethylformamide (DMF) (purity > 99.5%) and methanol (purity > 99.5%) were purchased from Fujifilm Wako Pure Chemical, Co., Inc., (Tokyo, Japan). Diethyl ether (purity > 95%), *cis*-aconitic anhydride (CAA; purity > 95%), dextran sulfate (M_r_ ~40,000), and 4-(2-Hydroxyethyl)-1-piperazineethanesulfonic acid (HEPES) (1.0 M), Dulbecco’s phosphate-buffered saline (D-PBS), fetal bovine serum (FBS), penicillin-streptomycin and RPMI-1640 medium were purchased from Sigma-Aldrich (St. Louis, MO, USA). Oxalyl Chloride (purity > 98%) and anhydrous dichloromethane (purity > 98%) were purchased from Tokyo Chemical Industry Co., Ltd. (Tokyo, Japan). The CT26 cells were obtained from Riken BioResource Center (Tsukuba, Japan).

### 2.2. Polymer Synthesis

The PEG-pLL block copolymer was prepared as previously reported [23]. PEG-pLL(TFA) block copolymer was firstly synthesized by ring-opening polymerization (ROP), taking MeO-PEG-NH_2_ (*M_w_* = 12,000 g mol^−1^) as the initiator. In brief, MeO-PEG-NH_2_ (1 g, 0.083 mmol) and Lys(TFA)-NCA (1 g, 3.75 mmol) were separately dissolved in 10 mL anhydrous DMF. The two solutions were then mixed under Ar flow and allowed to react in a 35 °C water bath for 48 h. The mixture was precipitated in diethyl ether to obtain PEG-pLL(TFA). GPC measurement was performed on the TOSOH HLC-8220 system (TOSOH, Tokyo, Japan) equipped with TSK gel G4000HHR and G3000HHR columns and an internal refractive index (RI) detector. PEG Standards ReadyCal kit (Sigma-Aldrich, Saint Louis, MO, USA) containing PEG with different molecular weights (range from ~250 to ~45,000 Da) was used for the calibration. Measurement was conducted at 40 °C in DMF containing 10 mM lithium chloride at a flow rate of 0.8 mL min^−1^. Polymers were firstly dissolved in DMF containing 10 mM lithium chloride at 1 mg/mL and filtered through RephilQuick Syringe Filter (PTFE, 13 mm, 0.45 µm) before GPC analysis.

Deprotection of the TFA group was completed by dissolving the collected PEG-pLL(TFA) in methanol with 1M NaOH and kept reacting in a 35 °C water bath for 12 h. The mixture was purified by dialysis against 0.01 M HCl and pure water (molecular weight cut-off (MWCO): 6000–8000 Da), then lyophilized to get the PEG-pLL. The degree of polymerization (DP) of the lysine groups was determined by ^1^H-NMR (400 MHz, JEOL ECS-400, JEOL, Tokyo, Japan) in D_2_O, and the polydispersity of the polymer was tested by the aqueous GPC (Extrema 4500 Model, JASCO) (eluent: D-PBS, pH 7.4; temperature: 25 °C; flow rate: 0.75 mL min^−1^; detector: UV 220 nm).

PEG-pLL(CAA) block copolymer was synthesized by further conjugating CAA molecules to the lysine block of the PEG-pLL via a condensation reaction between an acid chloride and amine. Briefly, CAA (153 mg, 1 mmol) was reacted with oxalyl chloride (2 mL, 2.5 g, 20 mmol) at 25 °C for 12 h to prepare the acid chloride of CAA (CAA-Cl). The CAA-Cl was purified by vacuum evaporation to totally remove the excess oxalyl chloride, and the product was collected as an oily liquid. PEG-pLL (200 mg, 0.011 mmol) was reacted with the prepared CAA-Cl at 25 °C in 10 mL anhydrous DMF for 12 h. The PEG-pLL concentration was controlled at 20 mg/mL. The final product, PEG-pLL(CAA), was obtained by precipitating the mixture in diethyl ether. The number of CAA units in PEG-pLL(CAA) was confirmed by ^1^H-NMR in DMSO-*d*_6_ at 80 °C.

### 2.3. In Vitro Transcribed mRNA

Plasmid RNA temples for preparing *Gaussian luciferase* (*gluc*) and *Firefly luciferase* (*fLuc*) mRNA were prepared by inserting corresponding protein-coding sequences having 120 bp poly A/T sequence into the pSP73 vector (Promega, Madison, WI, USA). Linearized *gluc* and *fluc* plasmids were used as temples for in vitro transcription using a mMESSAGE mMACHINE T7 Ultra Kit (Thermo Fisher Scientific, Waltham, MA, USA) to produce *gluc* and *fluc* mRNA. The obtained mRNA was then purified with the RNeasy Mini Kit (Qiagen, Hilden, Germany). The mRNA concentration was determined by measuring the absorbance at 260 nm using a NanoDrop 3300 spectrophotometer (Thermo Fisher Scientific). 

### 2.4. Micelle Preparation

To prepare PEG-pLL-based mRNA-loaded micelles (PEG-pLL/m), the synthesized mRNA (1000 ng) was dissolved in 10 mM HEPES buffer (pH 7.4) at 40 ng/µL. The PEG-pLL block copolymer was also dissolved in 10 mM HEPES buffer (pH 7.4) at 1 mg/mL, and a 4.5 µL polymer solution was then mixed with 20.5 µL 10 mM HEPES buffer (pH 7.4) for a final polymer solution. mRNA-loaded PEG-pLL/m were prepared by mixing polymer solution with 25 µL mRNA solution. The final molar ratio of amine groups in polymer (N)/phosphate groups in mRNA(P) (N/P ratio) was equal to 4, and the molar ratio of polymer to mRNA was 65. Micelles were then kept at 4 °C for 1 h before use.

To prepare the PEG-pLL(CAA)-based mRNA-loaded micelles (PEG-pLL(CAA)/m), the synthesized mRNA (1000 ng) and PEG-pLL(CAA) were dissolved in pH 8.5 and pH 3.5 10 mM HEPES buffer, respectively. Then, 7.5 µL PEG-pLL(CAA) solution (0.5 mg/mL) was mixed with 12.5 µL 10 mM HEPES buffer (pH 3.5), and the final polymer solution was then gradually added to 20 µL mRNA solution (50 ng/µL). The molar ratio of polymer to mRNA was fixed at 63, which is comparable to that of the PEG-pLL/m. The volume ratio of polymer solution to mRNA solution was controlled at 1:1. Finally, the pH of the micelle solution was adjusted to 7.4 by adding 10 mM HEPES (pH 8.5) and stirred for 2 h before use.

### 2.5. Micelle Characterization

The Z-average diameter of the micelle was measured by dynamic light scattering (DLS) using a Zetasizer Nano ZS (Malvern Instruments Ltd., Malvern, UK). mRNA loading was also confirmed by fluorescence correlation spectroscopy (FCS) measurement. Firstly, mRNA was labeled with Cy5 using a Label IT Tracker Intracellular Nucleic Acid Localization Kit (Mirus Bio Corporation, Madison, WI, USA). Using the Cy5-labeled mRNA, PEG-pLL/m and PEG-pLL(CAA)/m were prepared as described above. Micelle solution was then diluted to 2 mM Cy5-labeled mRNA equivalent concentration with 10 mM HEPES buffer (pH 7.4). The diluted micelle solution (200 μL) was then used for FCS measurement via He-Ne laser (633 nm) scanning with an LSM-780 confocal laser scattering microscope (CLSM; Carl Zeiss AG, Oberkochen, Germany). Herein, Alexa Fluor 647 dye (Thermo Fisher Scientific) was selected as a standard, and the diffusion coefficient of Cy5-labeled mRNA was obtained by comparing their diffusion time with that of Alexa Fluor 647. Moreover, the obtained count per molecule was analyzed to calculate the association mRNA number per micelle according to the following equation:

$$Association\;number=\frac{Count\;per\;molecule\;\left(Micelle\right)}{Count\;per\;molecule\;\left(Naked\;mRNA\right)\;}$$

### 2.6. Encapsulation Efficiency of mRNA

Encapsulation efficiency was measured by the fluorescence of the highly RNA selective Qubit RNA HS dye (Q32852, Invitrogen, Waltham, MA, USA). The free mRNAs amount was quantified because the dye shows fluorescence increases upon binding to free mRNAs but would not show fluorescence to micelle-loaded mRNAs due to lack of association. Briefly, micelles were prepared as described above using 250 ng of *Gluc* mRNA. This was added to 190 μL of Qubit RNA working buffer following the manufacturer’s protocol. After 10 min incubation, the fluorescence of the resultant solution was measured using an excitation wavelength of 630 nm and emission wavelength of 680 nm by a multimode microplate reader (Tecan Group Ltd., Zürich, Switzerland). The encapsulation efficiency was calculated using the below formula:

$$Encapsulation\;efficiency=\frac{Amount\;of\;feed\;mRNA-Amount\;of\;detected\;free\;mRNA\;}{Amount\;of\;feed\;mRNA}\times 100$$

### 2.7. Micelle pH Sensitivity 

To examine the pH sensitivity of the PEG-pLL(CAA)/m, Cy5-labeled *gluc* mRNA-loaded micelles were prepared and incubated in 10 mM HEPES buffer with 150 mM NaCl (final mRNA concentration: 5 ng/μL) at pH 7.4, 6.5, 5.5 and 4.5 at room temperature for 1 h. Samples were then analyzed by FCS to obtain the diffusion coefficient of the micelles to track the micelle dissociation. The increase in the diffusion coefficient indicates the micelles losing their integrity to release the Cy5-labeled mRNA. To compare the stability of the micelles, the diffusion coefficient at each pH point was normalized to the diffusion coefficient of the micelles at pH 7.4.

### 2.8. Micelle Stability against Polyanion

Micelle stability against counter polyanion exchange was measured by FCS after mixing the micelles with sodium dextran sulfate. Briefly, Cy5-labeled mRNA micelle solutions containing Cy5-labeled mRNA were mixed with sodium dextran sulfate at different S/P ([sulfate in dextran sulfate]/[phosphate in mRNA]) ratios (final mRNA concentration: 5 ng/μL). The resulting solution was then kept at room temperature for 1 h, and the diffusion coefficient of Cy5-labeled mRNA was obtained by FCS measurement as mentioned above.

### 2.9. Stability of mRNA in Micelles in FBS

To test the micelle stability against nucleases, micelles loaded with *gluc* mRNA were incubated in 10% FBS (final mRNA concentration was adjusted to 6.25 ng/μL) for 15 min at 37 °C. The resulting mRNA containing solution was cleaned by the RNeasy Mini Kit, then reverse-transcribed with the ReverTra Ace qPCR RT Master Mix kit Toyobo Life Science, Osaka, Japan). Finally, qRT-PCR analysis was performed by the 7500 Fast Real-Time PCR Instrument (Applied Biosystems, Bedford, MA, USA) using a primer pair for *gluc* mRNA (Forward; TGAGATTCCT GGGTTCAAGG, and Reverse; GTCAGAACACTGCACGTTGG). 

### 2.10. Cytotoxicity Assay

CT26 cells were cultured on 96-well plates (3000 cells per well) and incubated in RPMI containing 10% FBS and 1% penicillin/streptomycin under 5% CO_2_ at 37 °C. Next, the cells were washed twice with PBS; the medium was replaced with medium with varying concentrations of polymer (1 μg/mL, 3 μg/mL, 6 μg/mL and 25 μg/mL). After 24 h incubation, the cytotoxicity was evaluated by Cell Counting Kit-8 (CCK-8, Dojindo Molecular Technologies Inc., Tokyo, Japan) assay following manufacturer’s instruction. The cytotoxicity of the in vivo-jetPEI (Polyplus-transfection, Illkirch-Graffenstaden, France) at 0.01×, 0.1×, 0.3×, 0.6×, 2.5× and 5×, the recommended concentration (according to the manufacturer’s instructions), was determined in the same way.

### 2.11. In Vitro Uptake

The cellular uptake of the micelles was measured by CLSM. CT26 cells (10,000 cells/well) were seeded on an 8-well chambered borosilicate cover glass (Lab Tek) and incubated in RPMI containing 10% FBS and 1% penicillin/streptomycin under 5% CO_2_ at 37 °C. After 24 h, Cy5-labeled *gluc* mRNA and mRNA-encapsulating micelles (700 ng mRNA per well, relative fluorescence intensity: 400 [RFU]) were applied to CT26 cells. After another 6 h, cells were washed with PBS three times, and the cell nucleus was stained with 1% Hoechst 33342 solution (Dojindo Laboratories, Kumamoto, Japan) for 5 min before LSM imaging.

### 2.12. In Vitro Cellular Transfection

CT26 cells were cultured in RPMI containing 10% FBS and 1% penicillin/streptomycin with 5% CO_2_ at 37 °C. To evaluate *gluc* expression efficiency, cells were seeded on a 96-well plate (50,000 cells/well). After 24 h incubation, 500 ng of *gluc* mRNA, PEG-pLL/m, and PEG-pLL(CAA)/m containing 500 ng of *gluc* mRNA were then applied to the cells. mRNAs were also complexed with in vivo-jetPEI at 1.2 μL PEI/μg of mRNA and transfected into cells according to the manufacturer’s instructions. After another 24 h, a 50 μL culture medium was collected for luciferase assay using a Renilla Luciferase Assay System (Promega, Madison, WI, USA) and a GloMax 96 Microplate Luminometer (Promega, Madison, WI, USA).

### 2.13. In Vivo Transfection

To prepare the CT26 tumor model, 1 × 10^6^ CT26 cells were inoculated into the flank of female Balb/c mice (female, 6 weeks old; Charles River Laboratories Japan, Inc., Yokohama, Japan). After 10 days, the tumor size reached approximately 200 mm^3^. The mice were randomized into four groups and received intratumoral injections of 5 μg *fluc* mRNA, PEG-pLL/m, PEG-pLL(CAA)/m, and PEI polyplex containing 5 μg *fluc* mRNA. At 1, 9 and 24 h post-injection, mice were intraperitoneally injected with 200 μL 50 mg/mL luciferin solution; *fluc* expression was imaged after 10 min using IVIS Spectrum imaging system (SP-BFM-T1, PerkinElmer, Waltham, MA, USA).

### 2.14. Statistical Analysis

The results are presented as the mean value ± standard deviation (s.d.). Groups were compared by performing a two-tailed Student’s *t*-test or one-way ANOVA test in GraphPad Prism 8 (GraphPad Software, Inc., San Diego, CA, USA).

## 3. Results and Discussion

### 3.1. Synthesis and Characterization of Block Copolymer

PEG-pLL(TFA) block copolymer was successfully synthesized from ROP of Lys(TFA)-NCA, taking the terminal amine of MeO-PEG-NH_2_ as the initiator as described in a previous paper [23]. The polymer showed a narrow molecular weight distribution (*M_w_/M_n_* = 1.04) (Figure S1). The TFA groups were then cleaved by alkaline hydrolysis to get PEG-pLL, and the final product was characterized by ^1^H-NMR analysis (D_2_O; 25 °C). The DP of the lysine block was determined to be 46 units from^1^H-NMR by comparing the characteristic peaks of -O-CH_2_-CH_2_-O- (δ = 3.57–3.84 ppm) in the PEG block with the peaks of -CH_2_-CH_2_-CH_2_- (δ = 1.30–1.80 ppm) in the pLL side chain. Moreover, narrow molecular weight distribution was observed in aqueous phase GPC (Figure S1).

The resulting PEG-pLL block copolymer was then modified with CAA to produce PEG-pLL(CAA) by reacting CAA-Cl with lysine groups. The number of introduced CAA units was determined to be 15 from^1^H-NMR by comparing the peaks of -O-CH_2_-CH_2_-O- (δ = 3.46–3.61 ppm) in the PEG block with the characteristic peak of -CH- (δ = 5.61–5.74 ppm) in the CAA group (Figure S2).

### 3.2. Micelle Formation and Characterization

The PEG-pLL(CAA) block copolymer is designed to provide both ion complexation with mRNA through the amino groups and pH-sensitive covalent bonding between the amino groups and the CAA moieties. When the polymer is dissolved in acidic buffer (pH < 4), PEG-pLL(CAA) will remain in free polymer form with the protonated amines and the dehydrated anhydride structure. However, when mixing this polymer with mRNA in pH 8 buffer, the residual amine groups in the PLL block will complex with mRNA, and the CAA will react with the amines to cross-link the core of the micelles. The control PEG-pLL/m were assembled by mixing the PEG-pLL with *gLuc* mRNA in 10 mM HEPES buffer, as previously reported [14].

The size of the two micelles was examined by DLS. PEG-pLL and PEG-pLL(CAA) formed micelles of approximately 70 nm and 90 nm in diameter, respectively (Figure 1b and Table 1). In the following studies, FCS was applied for a detailed molecular characterization of the complexation behavior of mRNA with polymers because FCS is a powerful and versatile tool for nanocarrier characterization. In particular, FCS can monitor and quantify the size of the nanoparticles, drug loading efficiency, nanoparticle stability and possible interactions with plasma proteins as well as cargo release under physiologically relevant triggers [24]. First, the encapsulation of mRNA in the micelles was determined by FCS. The diffusion coefficient of free Cy5-labeled mRNA (19.95 ± 1.98 μm^2^/s) decreased after mixing with the block copolymers (12.07 ± 0.87 μm^2^/s for PEG-pLL/m, 10.94 ± 2.50 μm^2^/s for PEG-pLL(CAA)/m), which indicated the successful encapsulation of mRNA in the micelles. Moreover, by comparing the ratio of the counts per molecule between the micelles and Cy5-labeled mRNA, it was found that both PEG-pLL/m and PEG-pLL(CAA)/m have around 1 mRNA per micelle (Table 1). The encapsulation of the mRNA PEG-pLL(CAA)/m was close to 100% (Table 1).

The pH sensitivity of the micelles was also evaluated by FCS after incubating the micelles in 10 mM HEPES buffer with 150 mM NaCl with different pH. Here, to better understand the micelle disassembly process, the diffusion coefficient of micelles in the different pH buffers was normalized with the initial diffusion coefficient of each micelle. In the complexation of PEG-pLL(CAA) and Cy5-mRNA, the normalized diffusion coefficient increased from 1.05 to 1.76 when the pH decreased from 6.5 to 4.5, i.e., the endosomal pH range, which indicates PEG-pLL(CAA)/m gradually disassociated as a result of acidification. At pH 4.5, the diffusion coefficient of PEG-pLL(CAA)/m (19.11 ± 1.33 μm^2^/s) was close to that of naked mRNA (19.95 ± 1.87 μm^2^/s), indicating the complete release of mRNA. On the other hand, the normalized diffusion coefficient of PEG-pLL/m remained stable, indicating that the PEG-pLL/m kept associated with Cy5-mRNA when decreasing the pH (Figure 1c).

### 3.3. Stability of the Micelles

**Figure 2:**
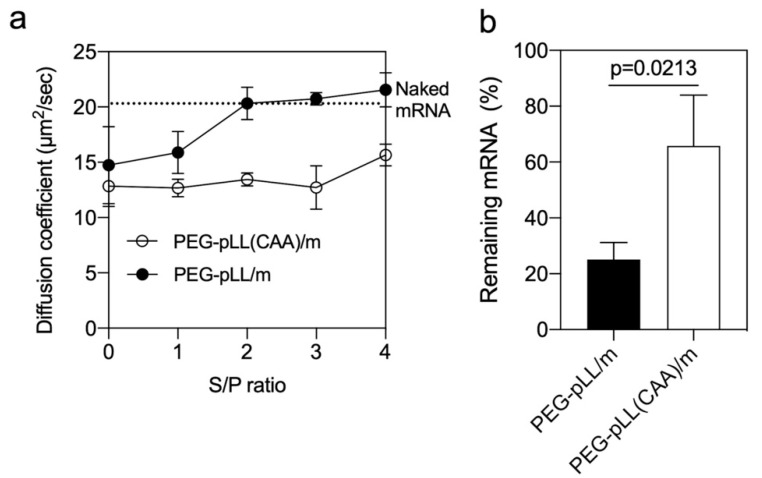
Stability of micelle. (**a**) Diffusion coefficient of micelles after incubation with sodium dextran sulfate at different S/P (sulfate in dextran sulfate/phosphate in mRNA) ratios for 1 h. The upper dashed line represents the diffusion coefficient of naked mRNA. Data were expressed as the mean ± s.d. (*n* = 10). (**b**) Remaining mRNA after incubating micelles with serum at 37 °C for 15 min. The mRNA amount was measured by qRT-PCR. Data were expressed as the mean ± s.d. (*n* = 3). *p*-value was calculated by the two-tailed Student’s *t*-test.

Counter polyanion exchange is one of the major concerns for nucleic acid delivery systems [11]. PIC-based micelles can be dissociated by polyanions in biological environments [25,26,27]. Thus, we evaluated the stability of the micelle in the presence of negatively-charged dextran sulfate at pH 7.4. The micelles were incubated at different sulfate in dextran sulfate/phosphate in mRNA (S/P) ratios for 1 h, and the diffusion coefficient of Cy5-labeled mRNA was measured by FCS. In the PEG-pLL/m, the mRNA was completely released from the micelles at S/P = 2. On the other hand, the diffusion coefficient of the PEG-pLL(CAA)/m remained stable even at S/P = 4 (Figure 2a), suggesting the ability of the cross-linked micelles to retain mRNA.

The capability of micelles to protect the loaded mRNA from enzymatic degradation was investigated by qRT-PCR after incubating with 10% FBS. The results showed that more than 66% of the mRNA was still detectable in PEG-pLL(CAA)/m compared to 25% of the mRNA in the PEG-pLL/m (Figure 2b). These results suggest that the core cross-linking by CAA significantly protected mRNA from polyanion and nuclease attacks, which demonstrates the advantage of the proposed pH-sensitive cross-linking strategy.

### 3.4. In Vitro Activity

**Figure 3:**
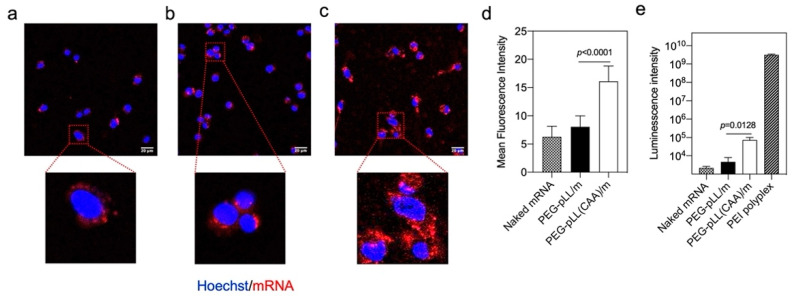
In vitro performance of micelles. Fluorescence in cultured CT26 cells with (**a**) naked Cy5-labeled mRNA, (**b**) PEG-pLL/m and (**c**) PEG-pLL(CAA)/m loading Cy5-labeled mRNA for 6 h incubation in medium, observed by confocal laser scanning microscopy (CLSM). Scale bars: 20 μm. (**d**) Cellular uptake efficiency was quantified from the mean fluorescence intensity of the pixels corresponding to Cy5 (*n* = 30 cells). (**e**) Efficiency of *gluc* protein expression in CT26 cells after 24 h incubation in CT26 cells. Data were expressed as mean ± s.d. (*n* = 3). Statistical significance was calculated by the two-tailed Student’s *t*-test.

The in vitro performance of mRNA micelles was studied in murine colon cancer cells (CT26). First, we confirmed the safety of the block copolymers by studying their in vitro cytotoxicity. After incubating CT26 cells with the PEG-pLL and PEG-pLL(CAA) polymers for 24 h, we found that both polymers are not cytotoxic at concentrations lower than 25 μg/mL (Figure S3a). Thus, polymer concentrations lower than 25 μg/mL were used in the following cellular studies. The cellular uptake was evaluated by CLSM using Cy5-labeled mRNA and PEG-pLL/m and PEG-pLL(CAA)/m loading Cy5-labeled mRNA. After 6 h incubation with the mRNA-based formulations, we found that the cellular uptake of mRNA was improved by the micelles (Figure 3). Moreover, the Cy5 signal in the cells treated with PEG-pLL(CAA)/m was 2-fold higher than that of PEG-pLL/m (Figure 3a–d). These results can be associated with the enhanced stability of the PEG-pLL(CAA)/m against polyanion exchange, as the proteoglycans on the cell membranes can disturb the PIC complexes through polyanion exchange, posing a major barrier to the internalization of carriers based on PIC assembly [28]. Thus, PEG-pLL(CAA)/m allowed higher stability against the polyanion exchange than PEG-pLL/m, resulting in a higher cellular uptake.

The ability of the micelles to produce proteins in the cells was evaluated by testing the luminescence levels after treatment with *gluc* mRNA-loaded micelles. Here, we used commercial PEI-based polyplexes as a positive control. The PEG-pLL(CAA)/m showed a significant 16-fold higher *gluc* expression than PEG-pLL/m (Figure 3e). These results can be associated with the enhanced capability of PEG-pLL(CAA)/m to protect mRNA from nuclease attack and promote intracellular delivery compared to PEG-pLL/m. Nevertheless, the in vitro transfection efficiency of the micelles was much lower than that of the positive control PEI-based polyplexes. It is worth noting that, while PEI exhibited high transfection efficacies, high cytotoxicity was also observed for PEI even at the recommended working concentration (Figure S3b), probably due to its strong membrane disrupting ability [29].

### 3.5. In Vivo Activity

**Figure 4:**
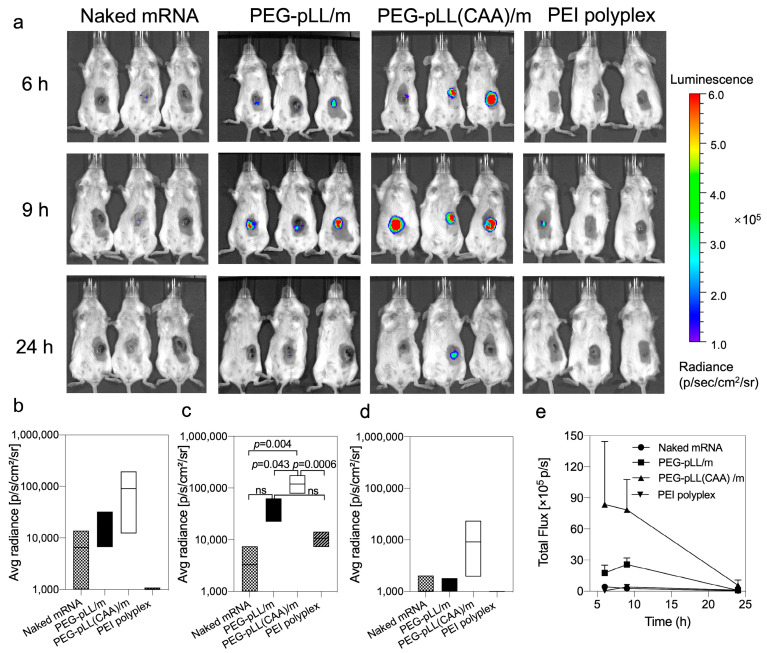
In vivo transfection of micelles. (**a**) Representative bioluminescence images following in vivo delivery of *fluc* mRNA in CT26 tumor-bearing mice. Mice were intratumorally injected with naked *fluc* mRNA, *fluc* mRNA-loaded PEG-pLL/m, *fluc* mRNA-loaded PEG-pLL(CAA)/m and PEI *fluc* mRNA polyplexes (5 μg mRNA per mouse, *n* = 3), and imaged at 6, 9 and 24 h post-injection. Quantification of luminescence signals from IVIS images at (**b**) 6 h, (**c**) 9 h and (**d**) 24 h post-injection. Data were expressed as the mean ± s.d. (*n* = 3). Statistical significance was calculated by the one-way ANOVA test. ns means not significant. (**e**) Quantification analysis of luminescent signals at indicated time points.

The in vivo mRNA transfection was explored in CT26 tumor-bearing mice. The mice received intratumoral injections of free mRNA, the micelles and PEI-based polyplexes (Figure 4). Both PEG-pLL/m and PEG-pLL(CAA)/m exhibited enhanced *fluc* expression in vivo compared to free mRNA and the PEI-based polyplexes. Moreover, the onset of the expression was earlier and higher for PEG-pLL(CAA)/m than for PEG-pLL/m, as the bioluminescence from PEG-pLL(CAA)/m-treated tumors was detectable at 6 h after injection (Figure 4a,b). Additionally, the luminescence signal from the tumors receiving PEG-pLL(CAA)/m was prolonged with a maximum at 9 h after injection (Figure 4a–d), which was significantly higher than that of the PEG-pLL/m group (Figure 4a,c). By calculating the area under the total flux/time curve from 6 to 24 h (AUC_6–24h_) (Figure 4d), we found that PEG-pLL(CAA)/m achieved 3.3-fold higher AUC_6–24h_ in the tumor than PEG-pLL/m. These results support the ability of pH-sensitive cross-linked micelles to improve the delivery of mRNA in vivo.

## 4. Conclusions

In this study, we successfully designed pH-responsive cross-linked micelles for mRNA delivery based on PEG-pLL(CAA). These micelles were stable at pH 7.4, while they dissociated to release mRNA when the pH was decreased below 6.5. The ability of the PEG-pLL(CAA)/m to remain stable at pH 7.4 allowed robust stability against polyanion exchange and nuclease attack. Thus, PEG-pLL(CAA)/m were able to promote intracellular delivery and gene expression in both in vitro and in vivo studies. Moreover, the PEG-pLL(CAA)/m provided higher gene expression in murine tumors compared to the commercial transfection reagent PEI. Our findings demonstrated the potential of the PEG-pLL(CAA)-based micelle system for mRNA delivery, confirming the feasibility of introducing pH-responsive covalent cross-linking for enhancing the in vivo performance.

## Figures and Tables

**Table 1:** Characteristics of PEG-pLL and PEG-pLL(CAA)-based micelles.

Samples	Size (nm) (Mean ± s.d.) ^(a)^	Polydispersity Index ^(a)^	Diffusion Coefficient (μm^2^/s) (Mean ± s.d.) ^(b)^	Count per Molecule (Mean ± s.d.) ^(b)^	Association mRNA Number in Micelles (Mean ± s.d.) ^(b)^	Encapsulation Efficiency ^(c)^
Naked mRNA	-	-	19.95 ± 1.98	5.82 ± 0.27	-	-
PEG-pLL/m	72 ± 1	0.29	12.07 ± 0.87	7.48 ± 0.79	1.36	100%
PEG-pLL(CAA)/m	94 ± 1	0.23	10.94 ± 2.50	9.85 ± 2.10	1.78	96%

^(a)^ Determined by DLS (*n* = 3). ^(b)^ Determined by FCS (*n* = 10). ^(c)^ Determined by Qubit RNA HS assay.

## Data Availability

The main data that support the findings of this study are available within the paper and its Supplementary Information.

## Supplementary Materials

The following supporting information can be downloaded at: https://www.mdpi.com/article/10.3390/pharmaceutics14061205/s1, Figure S1: (a) GPC chromatogram of PEG-pLL(TFA) (1 mg/mL, solvent: DMF with 10 mM lithium chloride, temperature: 40 °C, flow rate: 0.8 mL min^−1^) (b) ^1^H-NMR of PEG-pLL (polymer concentration: 10 mg/mL, solvent: D_2_O, and temperature: 25 °C), Figure S2: ^1^H-NMR of PEG-pLL(CAA) (polymer concentration: 10 mg/mL, solvent: DMSO-d6, and temperature: 80 °C), Figure S3: Viability of CT26 cells by CCK-8 assay after incubation with (a) polymers at 1 μg/mL, 3 μg/mL, 6 μg/mL and 25 μg/mL and (b) PEI at 0.01×, 0.1×, 0.3×, 0.6×, 2.5× and 5× the recommended concentration for 24 h. Error bars represent s.d. (*n* = 3 wells).
