# Supplementary material for: Polymeric Micelles with pH-Responsive Cross-Linked Core Enhance In Vivo mRNA Delivery
Source: Pharmaceutics. 2022 Jun 6;14(6):1205. doi: 10.3390/pharmaceutics14061205 (PMC9231146; doi:10.3390/pharmaceutics14061205)
Supplement: Supplementary file 1 [file pharmaceutics-14-01205-s001.zip › pharmaceutics-1721973-supplementary.pdf]

pharmaceutics

MDPI

Supplementary Materials: Polymeric micelles with pH-responsive cross-linked core enhance *in vivo* mRNA delivery

Wenqian Yang, Pengwen Chen, Eger Boonstra, Taehun Hong and Horacio Cabral *

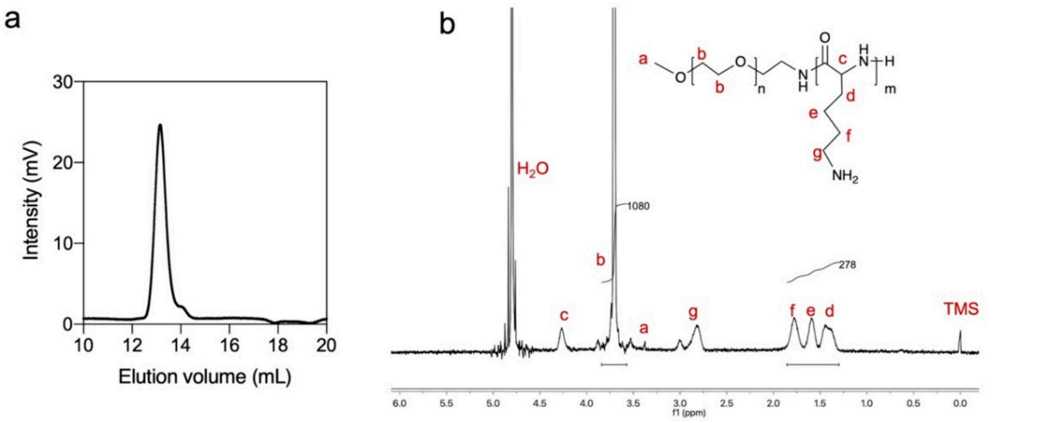

Figure S1. (a) GPC chromatogram of PEG-pLL(TFA) (1 mg/mL, solvent: DMF with 10 mM lithium chloride, temperature: 40 °C, flow rate: 0.8 mL min^{-1}) (b) ^{1}H-NMR of PEG-pLL (polymer concentration: 10 mg/mL, solvent: D_2O, and temperature: 25 °C).

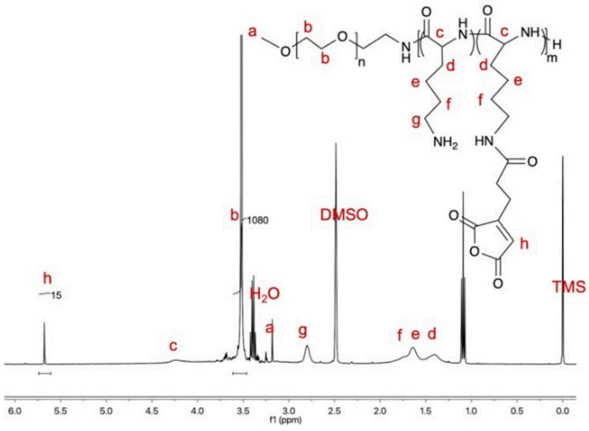

Figure S2. ^{1}H-NMR of PEG-pLL(CAA) (polymer concentration: 10 mg/mL, solvent: DMSO-d6, and temperature: 80 °C).

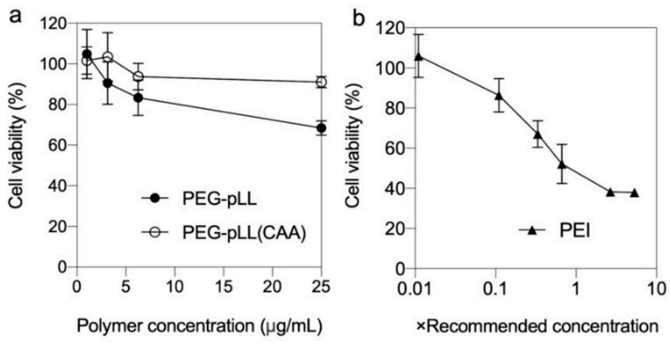

Figure S3. Viability of CT26 cells by CCK-8 assay after incubation with (a) polymers at 1 μg/mL, 3 μg/mL, 6 μg/mL and 25 μg/mL and (b) PEI at 0.01×, 0.1×, 0.3×, 0.6×, 2.5× and 5× the recommended concentration for 24 h. Error bars represent s.d. (n = 3 wells).
